# Transarterial embolisation of hepatocellular carcinoma with doxorubicin-eluting beads: single centre early experience

**DOI:** 10.2349/biij.6.1.e7

**Published:** 2010-01-01

**Authors:** O Nawawi, MN Hazman, BJJ Abdullah, A Vijayananthan, J Manikam, S Mahadeva, KL Goh

**Affiliations:** 1 Department of Biomedical Imaging, University of Malaya, Kuala Lumpur, Malaysia; 2 Department of Medicine, Faculty of Medicine, University of Malaya, Kuala Lumpur, Malaysia

**Keywords:** Drug-eluting beads, doxorubicin, embolisation, hepatocellular carcinoma

## Abstract

**Purpose:**

This is a retrospective study to evaluate the results of our early experience of using doxorubicin eluting beads (DEB) to treat patients with early and intermediate hepatocellular carcinoma (HCC).

Material and methods: A cohort of 19 patients (84.2% male; 15.8% female; mean age 59.2 years ± 11.0; range, 32-80 years) with documented HCC of size 1.8-10cm (mean, 4.0cm ± 1.8 ) undergoing DEB transarterial chembolisation (TACE) was reviewed. All patients had at least one image examination (multiphase computed tomography or magnetic resonance imaging) after embolisation.

**Results:**

A total of 32 procedures were performed. The objective response according to the European Association for the Study of the Liver criteria was 57.9% at 1-month, 42.8% at 6-month and 50.0% at 1-year follow up. There were 4 (21.1%) treatment-related complications (1 liver abscess, 2 pancreatitis and 1 tumour rupture) which resulted in 2 deaths. One death occurred 3 weeks after second embolisation, due to ruptured pancreatic pseudocyst, giving a 5.3% 30-day mortality rate. Another patient died 2 months after embolisation caused by tumour rupture. Eight patients received radiofrequency ablation after embolisation for residual or recurrent tumours. The 1-year survival rate in the DEB TACE only group was 80% while the 1- and 2-year survival rate in the group that received radiofrequency after DEB TACE was 85.7% and 100% respectively.

**Conclusion:**

DEB TACE is safe and effective in select group of patients. Survival may be improved when combined with other treatment modality.

## INTRODUCTION

Transarterial chemoembolisation (TACE) involves selective delivery of a chemotherapeutic agent such as cis-platin, doxorubicin, and mitomycin in an emulsion with viscous material such as lipiodol, followed by injection of an embolising agent into the vessel supplying the liver tumour. This procedure is recommended by the Society of Interventional Radiology (SIR) as the first line treatment of inoperable hepatocellular carcinoma (HCC) with preserved liver function [1]. The advantages of TACE are the ability to deliver high concentration of drugs to the tumour and reduction of serious systemic side effects such as cardiac toxicity, myelosuppression and alopecia compared with systemic chemotherapy. TACE functions by de-arterialisation of tumour by the embolic agents and selective delivery of chemotherapeutic agents to the tumour. Recent randomised trials and studies have shown statistical survival benefits and improved therapeutic efficacy of TACE in unresectable HCC over supportive care or systemic chemotherapy [2, 3, 4]

Recently, embolic microspheres that have the ability to sequester doxorubicin hydrochloride from the solution and release it in a controlled and sustainable fashion were introduced for intra-arterial injection. The embolisation particles are made from a unique drug-eluting technology i.e., doxorubicin-eluting beads (DEB), based on a hydrogel that has been modified with sulphonate group [5]. They are available in diameters ranging from 40 to 1200 µm designed to allow gradual release of chemotherapy over time, to prolong the contact time between cancer cells and to avoid damage of the hepatic microcirculation [6]. Selective delivery of the loaded beads into the feeding arteries leads to vessel lumen occlusion and ischaemia, while doxorubicin is gradually released locally, leading to tumour necrosis [5, 7, 8]. Several studies have shown that following DEB TACE, systemic levels of doxorubicin are reduced significantly when compared with conventional TACE [8, 9, 10]. DEB has been shown to elute doxorubicin over a sustained period of time resulting in greater tumour response [7].

In this paper, the authors present the results of their early experience treating 19 confirmed HCC patients using DEB. The study population includes patients with early and intermediate HCC (stage A and B). They also calculated the survival rate of patients who received radiofrequency ablation after DEB TACE.

## MATERIAL AND METHODS

### Patients

This study is a retrospective cohort study based on the analysis of 19 patients (16 male and 3 female, mean age 59.2 years ± 11.0) with HCC, treated with transarterial embolisation of doxorubicin-eluting DC beads (Biocompatibles UK, Surrey, UK) from February 2007 to October 2008 in a single centre. Following embolisation, all patients had at least one follow-up imaging with either multiphase CT or MRI. Diagnosis of HCC was either confirmed by biopsy or based on radiological findings and alpha-feto protein level according to the Barcelona criteria [11]. The inclusion criteria for treatment with doxorubicin loaded beads were as follows: patients with HCC who were not suitable for resection, liver transplantation or percutaneous ablation; patients who developed recurrence following resection, percutaneous ablation or conventional TACE and patients who have declined surgery or radiofrequency ablation. Liver function criteria for enrolment included bilirubin <51 µmol/L and liver enzymes (aspartate aminotransferase and alanine aminotransferase) < 270 IU/L.

The exclusion criteria for treatment with doxorubicin (DC) loaded beads were patients with: extrahepatic metastasis, portal vein invasion, portosystemic shunts, encephalopathy, gastrointestinal bleeding, contraindication for hepatic embolisation (impaired clotting tests, renal insufficiency/failure, sepsis and bleeding coagulopathy) and tumour burden >50% of the liver volume.

### Embolisation technique and surveillance imaging

All procedures were performed in the interventional radiology suite by interventional radiologists after informed consent for the procedure was obtained from the patients; approval of an ethics committee was not required. Prior to embolisation, angiography of the coeliac and hepatic artery was performed to determine the feeding arteries of the tumour, identify variation in the liver arterial supply and exclude portal venous shunting. Loading of the beads was done *in vitro* an hour before the beginning of catheterization. The loaded beads were then aspirated from the vial into a syringe filled with nonionic contrast medium. Three different sizes of DC beads were used, 100-300 μm, 300-500 μm and 500-700 μm. The diameter of the beads chosen depended on the calibre of the feeder, size of tumour and vascularity of the tumour. For small tumours measuring less than 3 cm, DC beads of 100-300 μm were used while DC beads of 300-500 μm and 500-700 μm were used for larger tumours. Once the feeding artery was identified and catheter was in placement, the loaded beads were infused slowly under fluoroscopic guidance. The injection of the loaded beads was performed as selective as possible using either a 4F diagnostic catheter (hepatic catheter or Yashiro) or 2.7F microcatheter (Progreat; Terumo). For diffuse or multifocal tumours, lobar or segmental embolisation was performed. The beads were injected distal to the origin of the gastroduodenal, right gastric and cystic artery. Pre-treatment coil embolisation of non target arteries was not performed in any of the patients. The embolisation endpoint was sluggish flow of the tumour feeder vessels. Intravenous analgesia and antiemetic were administered before and during the procedure.

Follow up imaging was performed 4 weeks after embolisation and every 3 months after that. Repeat embolisation was scheduled “on demand” basis, 2 to 4 weeks after follow up imaging if there was residual viable tumour deemed unsuitable for radiofrequency ablation or surgery.

All CT scan studies were performed with a 16-slice multidetector CT (Light speed, General Electric Medical Systems, USA and Siemen Somatom Sensation 16, Munich, Germany). CT examination was performed using a 5-phase protocol including a non-enhanced acquisition. Early arterial phase (delay 20 s), late arterial phase (delay 30 s), portal venous phase (delay 60 s) and delayed venous phase (delay 80 s) were obtained using 120 mL of contrast (Iopromide 300 mg I/L, Schering, Germany) at a rate of 4 mL/s. The images were acquired with slice thickness 1.25 mm, collimation 2.5 mm and table speed 7.5 mm per gantry rotation. In a few select patients, gadoxetic acid enhanced MRI using General Electric 1.5 Tesla Signa (GE Medical System, Milwaukee, Wisconsin) was performed.

### Outcome measures

Tumour response to procedure was evaluated according to the amount of tumour necrosis detected on CT or MRI follow-up imaging as recommended by European Association for the Study of the Liver Disease (EASL) [12]. The EASL acknowledges “viable areas” as those that “present enhancement” and “necrotic” as those that “do not present enhancement”. In the EASL criteria, complete response (CR) is defined as complete disappearance of all known disease and no new lesions are seen; partial response (PR) when a 50% reduction in all tumoural area of all measurable lesions is present; stable disease (SD) in all other cases and progressive disease (PD) when there is 25% increase in size of one or more measurable lesions or if new lesions appear. Objective response (OR) included both a complete and partial response. Patients who underwent other treatment modalities (radiofrequency ablation, conventional TACE, surgical resection) following DC beads embolisation were analysed separately. The authors considered a complication that occurred within 4 weeks of the embolisation as procedure-related complication.

### Statistical analysis

Statistical analysis was performed using SPSS version 17. Values for all continuous variables are quoted as mean, standard deviation, minimum and maximum throughout. The paired t-test was used to demonstrate changes in biochemistry over time.

## RESULTS

### Patients

Table 1 tabulates baseline characteristics, response and survival of patients analysed in this series. Of the 19 patients that underwent the procedure, 12 (63.2%) patients presented with Child-Pugh score A and 7 (36.8%) with Child-Pugh score B. Fifteen (78.9%) patients had liver cirrhosis out of which 2 had hepatitis C virus (HCV)-related cirrhosis, 8 had hepatitis B virus (HBV)-related cirrhosis, 1 had alcohol-induced cirrhosis and 4 had cryptogenic cirrhosis. The patients were also staged according to the Barcelona Clinic Liver Cancer (BCLC) Staging system with 4 (21.1%) of 19 patients presenting with early stage (A) and the rest (78.9%) presenting with intermediate stage (B) [12].

**Table 1:** Baseline characteristics, response and survival of patients.

**Patient**	**Age**	**Sex**	**BCLC stage**	**Aetiology of HCC**	**Number of lesions**	**HCC size (mm)**	**Number ofDEB-TACE received**	**Other treatments after DEB-TACE**	**Response at 1/6/12 months (m)**	**Survival (months)**
1	65	Male	B	Cryp Cirr	2	50, 100	2		PD / PR / PD	12 m, then lost to f/up
2	54	Male	B	Hep B	1	95	2		SD / - / -	3 m, died due to pseudocyst rupture
3	64	Male	A	Hep B	1	26	2	RFA	PR / SD / -	8 m, on f/up
4	61	Male	B	Hep B	7	20 - 43	3		SD / SD / PR	18m, died due to progressive liver disease
5	80	Female	A	Hep C	1	18	2		PR / PR / CR	12m, on f/up
6	50	Male	A	Hep C	1	35	1	RFA	PR / CR / CR	15m, on f/up
7	67	Male	B	Hep C	1	70	2		SD / SD / -	10m, then lost to f/up
8	73	Female	B	Cryp Cirr	1	63	1		PR / CR / CR	14m, on f/up
9	61	Male	B	Hep B	4	20 - 35	1		CR / CR / CR	18m, on f/up
10	60	Male	B	Cryp Cirr	12	25 - 30	1	RFA	PD / PD / -	11m, died due to disease progression
11	66	Male	B	Alc Cirr	8	25 - 35	1		PR / - / -	2m, died due to tumour rupture
12	58	Male	B	Cryp cirr	2	16, 120	1		PD / - / -	1m, then lost to f/up
13	32	Male	B	Hep B	4	24 - 42	1	Surgery	CR / PD / PD	14 m, on follow up
14	38	Male	A	Hep B	1	37	1		CR / CR / CR	18m, on f/up
15	52	Male	B	Hep B	4	42 - 63	3	RFA	PR / PD / PD	19.5m, on f/up
16	64	Male	B	Hep B	4	31 - 44	1	RFA	PR / PR / PR	19m, on f/up
17	59	Male	B	Hep B	2	28, 50	1	RFA	PD / PR / SD	18m, on f/up
18	61	Male	B	Hep B	4	32 - 50	2		SD / PD / PD	16m, on f/up
19	59	Female	B	Hep B	2	60, 60	2	RFA	CR / PD / SD	27.5m, then lost to f/up

Cryp Cirr: Cryptogenic cirrhosis; Alc Cirr: Alcohol induced cirrhosis

CR: Complete Response; PR: Partial Response; SD: Stable Disease; PD: Progressive Disease

Seven of the 19 patients presented with single tumour while the rest had multicentric disease. Of the 12 patients that presented with multicentric disease, 9 patients had less than 5 lesions, 2 had 5 to 10 lesions and 1 patient had 12 lesions. The total number of lesions was 62 and the mean tumour diameter was 4.0 ± 1.8 cm. Eleven (57.9%) patients were found to have unilobar disease with right lobe involvement seen in 10 of them. The rest (42.1%) of the patients presented with bilobar disease.

### Procedural

A total of 30 embolisations were performed. All procedures were technically successful with no intraprocedural complication encountered. Ten patients (52.6%) received single embolisation, 7 patients completed 2 embolisations and 2 patients completed 3 sessions. A total of 32 vials of DC beads were used (mean, 1.7 vials per patient) with a maximum dose of 150 mg (range: 50 mg to 150 mg) per session. Radiofrequency ablation (RFA) was performed on 8 patients following embolisation for residual or recurrent tumour. Two patients received 2 RFA sessions after embolisation. A total of 10 RFA sessions were performed.

### Biochemical

Periprocedural laboratory test showed no statistically significant change in the level of bilirubin and gamma-glutamyl transferase after the procedure compared to the baseline. However, there was significant rise of alanine aminotransferase (*p* = 0.03) and aspartate aminotransferase (*p* = 0.002) 1 to 3 days after embolisation which returned to baseline 3 to 5 weeks after the procedure.

### Tumour response

**Figure 1:**
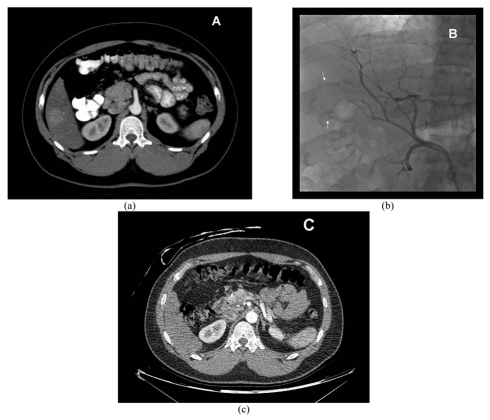
Multiphase CT in arterial phase showing a focal enhancing lesion in segment 6 (A). Angiogram before embolisation shows tumour (arrows) supplied by the branch of right hepatic artery (B). Multiphase CT in arterial phase 1 month after embolisation reveals complete disappearance of tumour, in keeping with complete response (C).

**Figure 2:**
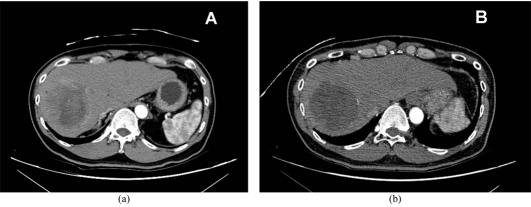
Multiphase CT in arterial phase showing a heterogenously enhancing lesion in the right lobe of liver (A). 1-month follow up CT in arterial phase shows tumour necrosis of most of the tumour with a small residual nodule at the periphery, in keeping with partial response (B).

Table 2 reports the tumour response according to the EASL assessment criteria of patients treated with only DEB TACE at 1-month, 6-month and 12-month follow-up. Figure 1 and 2 illustrate a case of complete response and partial response, respectively.

**Table 2:** Tumour response based on EASL criteria at 1-, 6- and 12-month follow up.

**Outcome**	**1-month follow upN (%)**	**6-month follow upN (%)**	**12-month follow upN (%)**
CR	4 (21.1)	3 (21.4)	3 (30.0)
PR	7 (36.8)	3 (21.4)	2 (20.0)
SD	4 (21.0)	2 (14.3)	0 ( 0.0)
PD	4 (21.1)	4 (28.6)	3 (30.0)
OR	11 (57.9)	6 (42.8)	5 (50.0)
Death	0 ( 0.0)	2 (14.3)	2 (20.0)

CR: Complete Response; PR: Partial Response; SD: Stable Disease; PD: Progressive disease; OR: Objective response

At 1-month follow-up, complete response was seen in 4 of the 19 patients (21.1%) and partial response was documented in 6 patients (31.5%). Five patients (26.3%) showed stable disease and 4 patients (21.1%) showed progressive disease. Objective response was seen in 52.6% of patients. There was no mortality recorded within 30 days of the embolisation.

At 6-month follow up, 1 patient was lost to follow up (patient 12) and 4 patients underwent RFA (patient 3, 6, 10, 16). Hence, 14 patients were available for the 6-month follow up. Six patients had completed 2 sessions of embolisation, with a treatment-related death recorded in one of the patients (patient 2). The death, which occurred 3 weeks after the second embolisation, was caused by cardio-circulatory collapse due to a ruptured pancreatic pseudocyst. Another patient died 2 months after one embolisation believed to be caused by tumour rupture (patient 11). The ruptured tumour showed partial response at the 1-month follow up CT scan. Objective response was documented in 6 (42.8%) of 14 patients with complete response seen in 3 patients and partial response recorded in 3 patients. One of the patients (patient 15) classified as having progressive disease by EASL criteria showed partial response of the initial target lesion but developed new lesion in the non targeted part of the liver.

At 12-month follow-up, another patient (patient 7) defaulted follow up and an additional 3 patients (patient 15, 18 and 19) underwent RFA. Sustained complete response was seen in 3 (30%) of 10 patients and sustained partial response seen in 2 (20%) patients. Survival at 12 months was 80%.

One patient underwent liver resection of new tumour lesion at 13 months (patient 13). One patient (patient 4) died at 18 months due to disease progression. No patient was available for the 24-month follow up. The mean duration of follow-up for patients who received DEB TACE only was 12.9 months ± 6.1 (range, 1 - 18 months).

Seven patients (36.8%) in this series received RFA after the first embolisation while 1 patient (5.2%) was ablated after the second embolisation, for residual or recurrent tumour. For these patients, the 1- and 2-year survival rate following the first embolisation was 85.7% (6 of 7) and 100% (1 of 1), respectively. One patient (patient 12) who underwent 2 sessions of RFA post embolisation succumbed to the disease 11 months from the time of embolisation. There was no treatment-related complication in this group of patients. The mean duration of follow up was 17 months ± 5.9 (range, 8 to 27.5 months).

### Side effects and complications

Pancreatitis resulting in treatment-related mortality was observed in one patient (patient 2) with intermediate HCC, after the second embolisation. The pancreatitis, which occurred 5 days after the embolisation and managed conservatively, was complicated by pseudocysts. Unexpectedly, one of the pseudocysts ruptured causing the patient to go into cardio-circulatory collapse. The death was the only 30-day mortality in this series. Another patient (patient 14) was retrospectively diagnosed to have pancreatitis after the 6-month follow up MRI revealed a 5 cm pancreatic pseudocyst. On further questioning, the patient gave a history of having intermittent ‘indigestion-like’ symptoms 3 months after the embolisation, which gradually worsened in the subsequent months. The pancreatic pseudocyst was then percutaneously aspirated under ultrasound guidance and the patient was completely asymptomatic following the aspiration.

One death (patient 11) occurred 8 weeks after embolisation, believed to be caused by rupture of the largest tumour, giving a treatment-related death of 10.5%. The tumour that ruptured measured 6 cm in diameter and was radioablated 5 times before the DEB TACE procedure.

Liver abscess was recorded in 1 patient (patient 13), 2 months after the second embolisation. The collection was successfully drained percutaneously but the patient underwent a resection 4 months later after a follow up CT revealed a recurrence at the margin of the resolving liver abscess. Overall, the total number of complications was 4 out of 19 patients (21.1%).

Post embolisation syndrome defined as fever, nausea, vomiting or abdominal pain was observed in all patients following the first procedure and 4 of 9 patients following the second session. The maximum hospital stay for post embolisation syndrome was 7 days. The median hospital stay was 1 day. There was no doxorubicin systemic toxicity (alopecia, marrow suppression or cardiac failure) documented in these patients.

## DISCUSSION

HCC is the fifth most common cancer globally with over 600,000 new cases diagnosed worldwide each year [13]. The incidence in developing countries is two to three times higher than in developed countries [14]. In Malaysia, HCC is among the ten most common cancers in the male population with a distinct predilection for Chinese [15, 16]. It is the leading cause of death in cirrhotic patients. Surgery, as a curative option carries significant perioperative mortality and morbidity in patients with cirrhosis and is indicated only in patients with single HCC without portal hypertension and preserved liver function [17, 18]. In patients with HCC that is not suitable for curative treatment, TACE represents the first-line approach that can improve 1- and 2-year survival [3, 11, 19]. The choices of chemotherapeutic agent and embolisation material may vary from centre to centre, however, the common denominator of this procedure is the intra-arterial injection of emulsified chemotherapeutic agent with viscous agent. The aim of the procedure is to deliver chemotherapeutic agents to the tumour selectively, and at the same time to induce ischaemic necrosis of the tumour.

Drug-eluting beads are new embolic agents for TACE that can be loaded with chemotherapeutic agent and designed to allow gradual release of chemotherapeutic agent locally and reduce the systemic toxicity of chemotherapeutic agent. *In vivo* study has documented that animals treated with DEB showed significantly lower plasma concentration of doxorubicin compared with control animals treated with doxorubicin intraarterially. This suggests higher tumour retention of doxorubicin in animals treated with DEB [19]. The high affinity for doxorubicin and the slow-release mechanism are unique properties of DEB that were not observed with other commercially available embolisation agents [5]. Varela et al. conducted the first human trial on doxorubicin loaded DC Beads, on Child-Pugh A cirrhotic patients with large or multifocal HCC. They confirmed that peak plasma doxorubicin level was lower with DEB compared with that of doxorubicin-lipiodol emulsion [10].

As with previous DEB TACE studies, there was no systemic toxicity from doxorubicin observed in this study [9, 10, 20, 21]. The total incidence of complication observed in this series was 12.5% per procedure or 21.1% per patient, comprising of 2 pancreatitis, 1 liver abscess and 1 tumour rupture. Two of the complications resulted in 10.5% of treatment-related death. The incidence of serious treatment-related complication and treatment-related death following conventional TACE had been reported to be 27.5% and 9.4%, respectively [4, 22]. Earlier investigators of DEB TACE had documented major complication rate of 7.4% and 42.9% and treatment-related death of 14.3% in their series [8, 23]. Overall, the complications encountered in this cohort are known complications of TACE that are reported to range from 0% to 50% [24].

Fatal outcome due to tumour rupture following TACE has been documented, usually in patients with large subcapsular tumours, as was the case in the authors' patient [25, 26]. Increased intratumoural pressure as a result of tumour necrosis or vascular injury secondary to embolisation has been thought to be the mechanism of tumour rupture following TACE [25].

Pancreatitis is an uncommon complication following embolisation, but has a significant morbidity and mortality potential if associated with local or systemic complications [27]. Clinically evident acute pancreatitis after embolisation occurs at an incidence between 1.7% and 4% but typical laboratory findings consistent with pancreatitis is shown in 40% of patients [28, 29]. The diagnosis of acute pancreatitis following embolisation can be missed as it can clinically mimic postembolisation syndrome, as was the case in the authors' patient. Hence, careful monitoring of serum pancreatic enzymes should be employed in cases of abdominal pain following TACE [27]. The proposed mechanism of pancreatitis following TACE is inadvertent embolisation through collateral vessels or regurgitation of embolic particles or chemotherapeutic agents to the non targeted arteries [30, 31]. Similar causative factors are used to explain a variety of other complications related to hepatic intra-arterial treatment especially to the gastroduodenal region. Acute cholecystitis attributed to inadvertent embolisation of the cystic artery was reported following DEB TACE [20]. In order to avoid serious adverse effects or damage to the gastroduodenal territory due to misdistribution of embolic or chemotherapeutic agents, embolisation of the gastroduodenal artery can be performed prior to embolisation [32, 33]. Prophylactic embolisation of nontarget arteries is a well-established approach to protection of non target organs in ^90^Y SIR-Spheres embolisation [34]. In view of the high chemoembolic mechanism of action of DEB and documented incidence of fatal pancreatitis in this series, similar preventive measures may be taken to avoid repeat incidence of fatal complications of non target organs, especially in cases where superselective injection are not possible.

Liver abscess is a well known DEB TACE related complication which had contributed to treatment-related mortality [2, 10, 23]. The 5.3% incidence of liver abscess in this series is comparable to previous studies which ranged between 1.6% to 14.2% [10, 20, 23]. The most important predisposing factor for this complication is the presence of biliary abnormality prone to ascending biliary infection such as bilio-enteric anastomosis, bilio-enteric fistula, endoscopic papillotomy and percutaneous biliary drainage [35]. These risk factors were was not present in the authors' patient (patient 13). Abscess formation after conventional TACE of 1.2% to 2% seems lower than after DEB TACE [1, 35, 36]. The reason for this observation could be due to the fact that unlike gelfoam particles used in conventional TACE, the beads used in DEB TACE are non-reabsorbable and can cause permanent ischemic damage to the liver [23].

The authors adopted the EASL criteria as their choice of monitoring treatment response as they take into account the development of necrosis, which better describe the effect of treatment. The parameter of necrosis is not taken into account in the Response Evaluation Criteria in Solid Tumours (RECIST), which measures the size of treated lesion. It is acknowledged that it is common for liver tumours to liquefy without significant change in total lesion diameter within short follow-up periods and that extensive tumour necrosis may not be paralleled by a reduction in the diameter of the lesion [11, 37].

The authors observed an objective response that ranged from 42.8% to 57.9% across 1 year (Table 2). Sustained objective response was recorded in 5 (50%) at 12-month follow up. These values are slightly lower than previous DEB TACE studies but higher than conventional TACE clinical trials of 16-35% [2, 4, 10, 20, 22, 38, 39, 40]. Malagari et al. in their DEB TACE trials on unresectable HCC reported objective response rate of 59.6% to 80.7% [20]. However, unlike the authors' series where patients with multicentric disease make up 63.2% of the study population, only patients with solitary tumour were included in their study [20]. One patient with complete response of the initial target lesion developed new lesions at the non targeted part of the liver at 6-month follow up and, hence, classified as having progressive disease (patient 19). These new lesions were either microscopic foci undetected by imaging at the time of recruitment or new multicentric tumours. Such lesions were probably not covered during treatment due to the selective delivery of DEB [38].

The 1-year survival rate of 80% in this study is similar if not higher than that of conventional TACE. In TACE studies where lobar embolisation was performed in 49% of the cases, the 1-, 2-, and 3-year survival rates were reported at 57, 31 and 26%, respectively [2]. In another paper where segmental TACE was applied in relatively small cancers which are potentially suitable for RFA, survival was 80%, 43%, and 23% at 1, 3 and 5 years, respectively [41]. Comparatively, the survival rate of patients who presented with single small HCC (< 4 cm) in the authors' series was 100% at 1-year. These were patients who had refused surgery, or who presented with new lesion after a successful RFA. Not surprising this small cohort of patients also achieved 100% objective response across 1 year.

A total of 42% of patients in the authors' series underwent RFA after DEB TACE. In view of the considerably high rate of patients in this group the authors have also included the survival analysis of this cohort. The 1-year survival rate of 85.7% (following the first embolisation) was slightly higher than that of the DEB TACE only group. At the time of writing, only 1 death was recorded at 11 months after the first embolisation. The longest surviving patient in this study belonged in this group. The patient survived 27.5 months after embolisation and was in partial response at 24-month follow up. Improved survival time had been reported in patients with early HCC treated with resection or TACE followed by RFA as compared to patients receiving TACE alone [42]. Early literature on patients with unresectable HCC treated with RFA after TACE reported promising mid-term clinical success with projected 1- and 2-year survival rate of 89.7% and 67.1%, respectively [43]. However, the overall usefulness of this combined therapy has yet to be established by large series and risk-benefit analysis.

In summary, the review of the authors' early experience shows that DEB TACE is well tolerated and effective in treating patients with early and intermediate HCC. When combined with other treatment modality, the survival time can be prolonged.
